# Supplementary material for: Preparation and Characterization of a Glutathione-Responsive Doxorubicin Prodrug Modified by 2-Nitrobenzenesulfonamide Group—Its Selective Cytotoxicity Toward Cells with Enhanced Glutathione Production
Source: Int J Mol Sci. 2025 Apr 26;26(9):4128. doi: 10.3390/ijms26094128 (PMC12071201; doi:10.3390/ijms26094128)
Supplement: Supplementary file 1 [file ijms-26-04128-s001.zip › ijms-3464189-supplementary.pdf]

Supplementary Materials

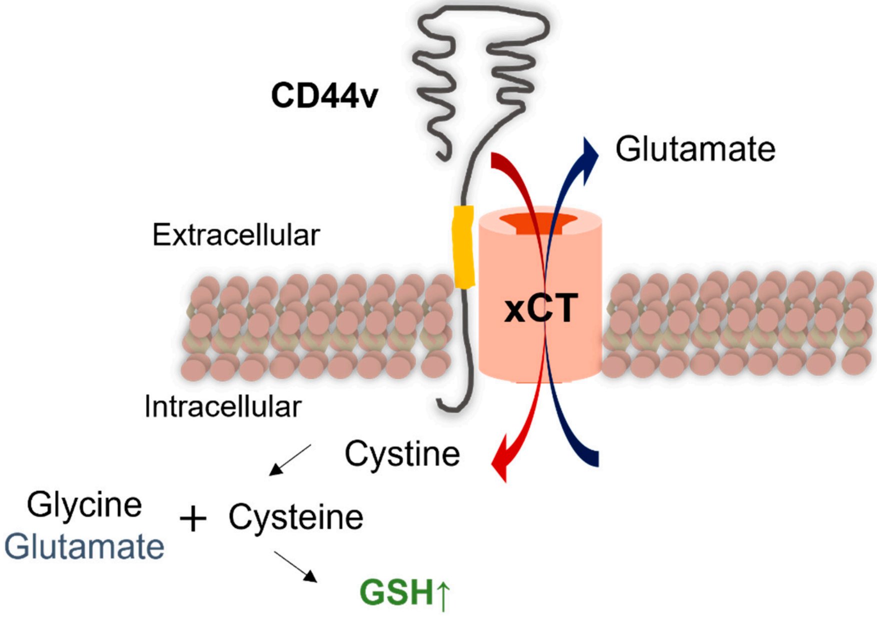

Figure S1: CD44v-mediated antioxidant system in cancer cells.

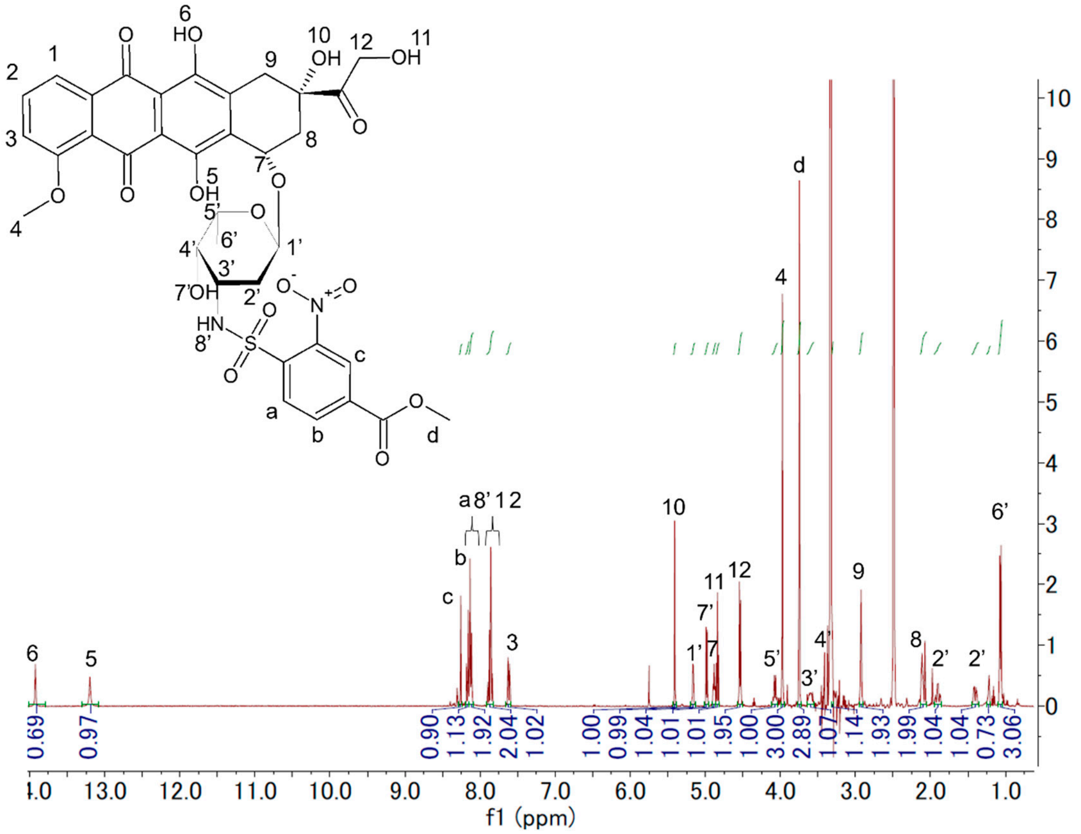

Figure S2: Structure determination using ^1H NMR (400 MHz, DMSO-d_6) of Ns-Dox.

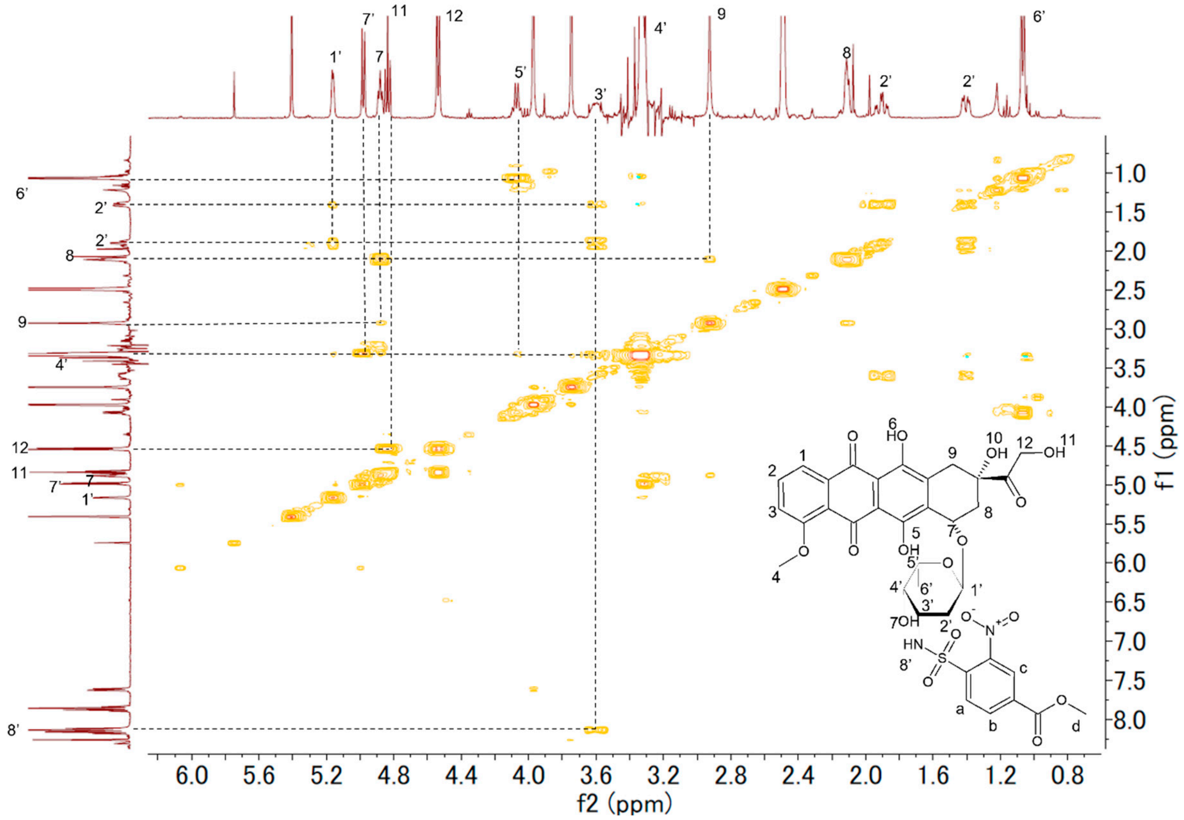

Figure S3: Structure determination using ^1H-^1H COSY NMR (400 MHz, DMSO-d_6) of Ns-Dox.

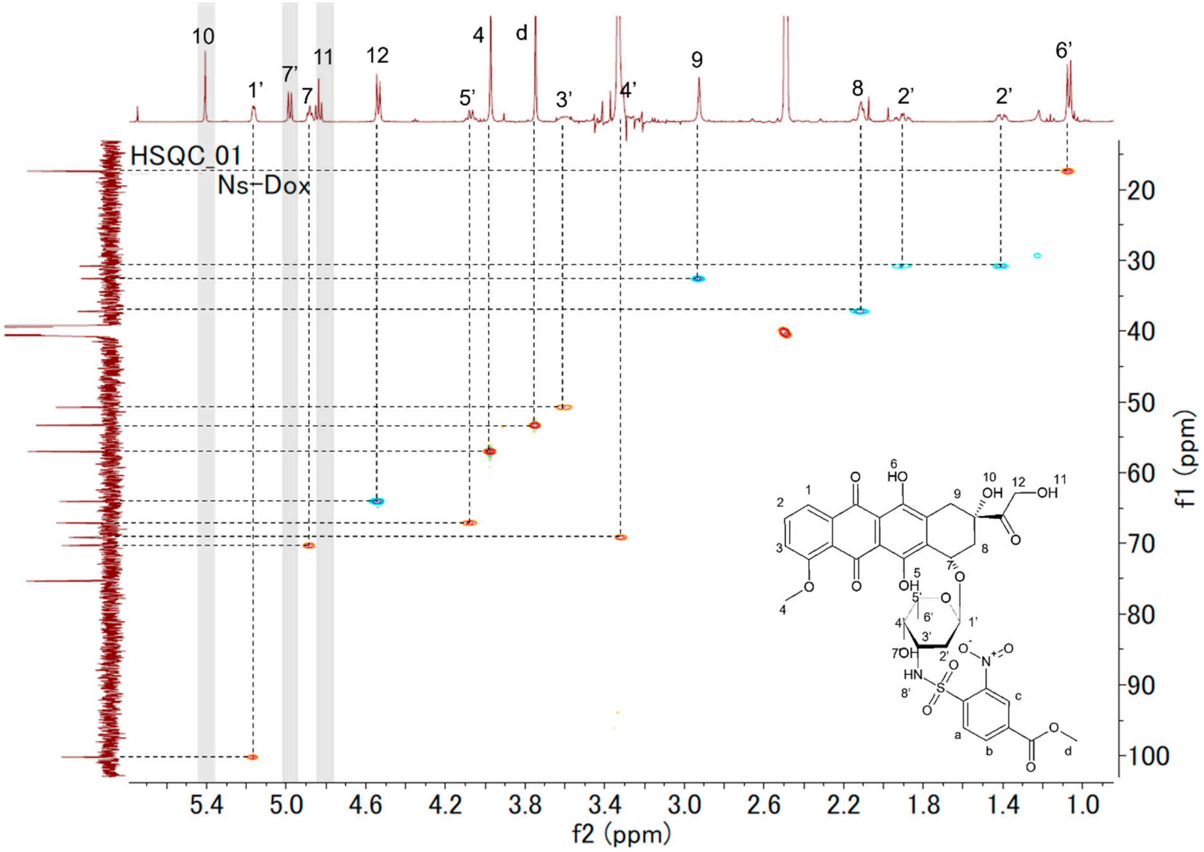

Figure S4: Structure determination using ^{13}C-^{1}H HSQC NMR (400 MHz, DMSO-*d*$_6$) of Ns-Dox.

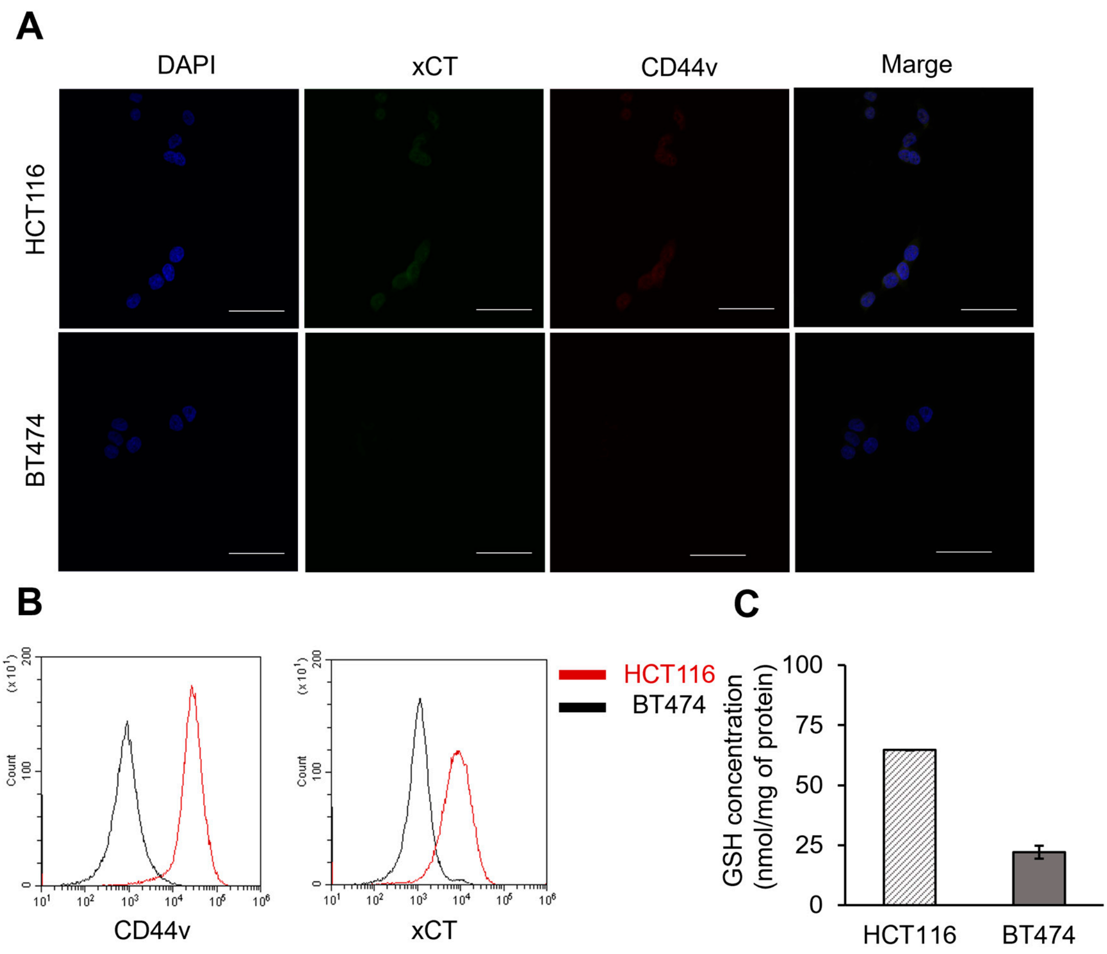

Figure S5: Immunohistochemical staining of HCT116 and BT474 cells for surface antigens CD44v-9 and xCT and their GSH concentrations were conducted. (A) The immunofluorescence analysis was performed using confocal laser scanning microscopy with Texas Red: λ_{ex}=561 nm, λ_{em}=570−670 nm (CD44v-red), Alexa Fluor 488 λ_{ex}=488 nm, λem=500−540 nm (xCT-green), DAPI λ_{ex}=405 nm, λ_{em}=430−470 nm (Nuclear-blue). 50 μm scale. (B) Immunofluorescence analysis using flow cytometry Laser: Blue FITC λ_{ex}=488 nm, λ_{em}=525/40 nm. (C) Intracellular GSH concentration per protein in HCT116 and BT474 cells. The absorbance was measured at 405 nm.

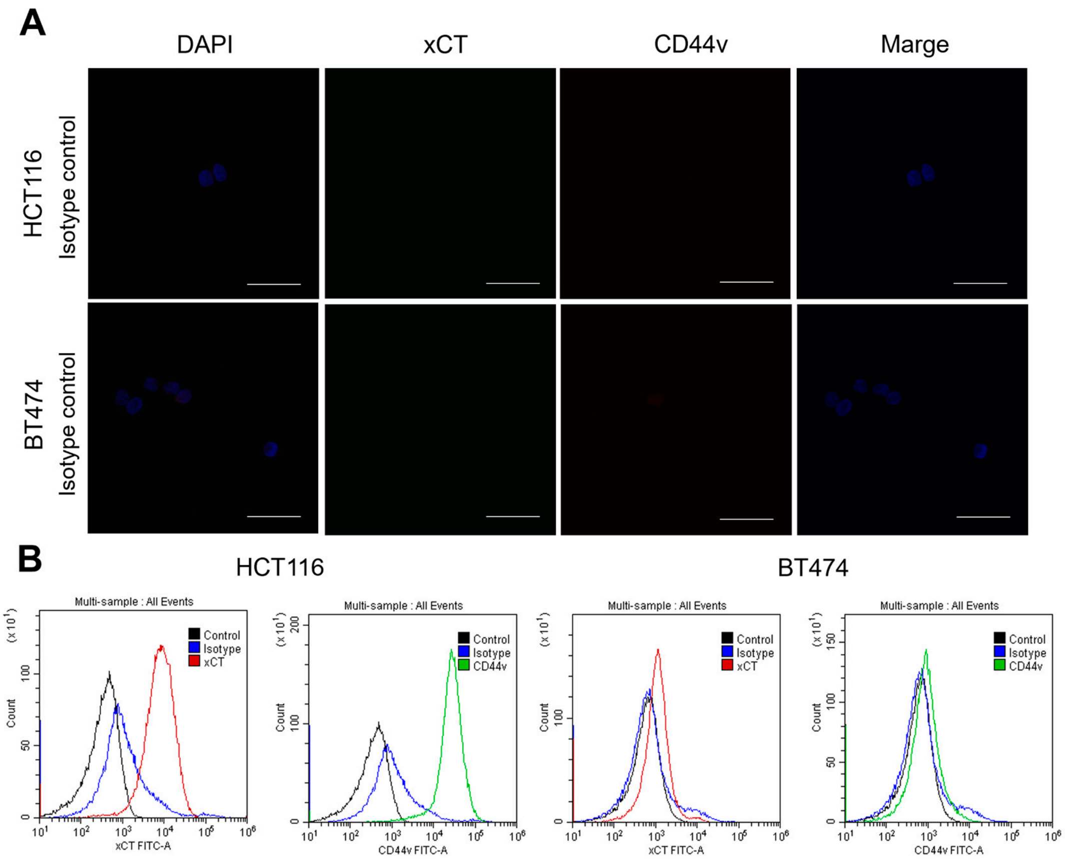

Figure S6: Isotype controls were used to confirm the antibody specificity of CD44v and xCT. (A) The immunofluorescence analysis was performed using confocal laser scanning microscopy with Texas Red λ_{ex}=561 nm, λ_{em}=570−670 nm (CD44v-red), Alexa Fluor 488 λ_{ex}=488 nm, λ_{em}=500−540 nm (xCT-green), DAPI λ_{ex}=405 nm, λ_{em}=430−470 nm (Nuclear-blue). 50 µm scale. (B) Immunofluorescence analysis using flow cytometry Laser: Blue FITC λ_{ex}=488 nm, λ_{em}=525/40 nm.

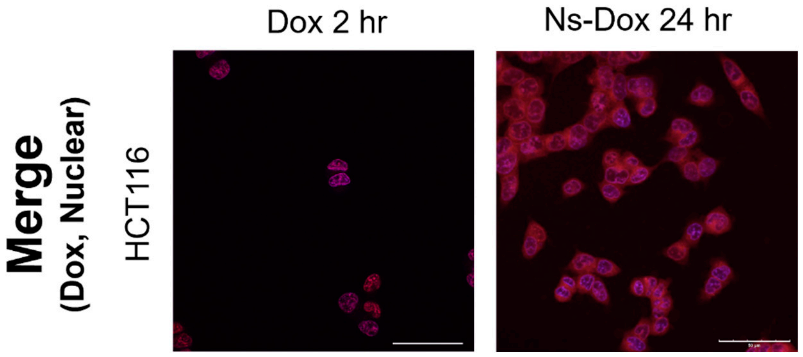

Figure S7: Comparison of HCT116 cells 2 hr after addition of Dox 20µM and 24 hr after the addition of Ns-Dox 20µM using confocal laser microscopy. Alexa Fluor 594 (Dox-red) λ_{ex}=561 nm, λ_{em}=570−670 nm, (Nuclear-blue) λ_{ex}=405nm, λ_{em}=430−470 nm, scale 50 µm.
